# Spleen extracellular matrix provides a supportive microenvironment for β-cell function

**DOI:** 10.22038/IJBMS.2022.65233.14360

**Published:** 2022-09

**Authors:** Layasadat Khorsandi, Mahmoud Orazizadeh, Darioush Bijan Nejad, Abbas Heidari Moghadam, Fereshteh Nejaddehbashi, Yousef Asadi Fard

**Affiliations:** 1 Cellular and Molecular Research Center, Medical Basic Sciences Research Institute, Ahvaz Jundishapur University of Medical Sciences, Ahvaz, Iran; 2 Department of Anatomical Sciences, Faculty of Medicine, Ahvaz Jundishapur University of Medical Sciences, Ahvaz, Iran; 3 Department of Anatomical Sciences, School of Medicine, Dezful University of Medical Sciences, Dezful, Iran

**Keywords:** Artificial organs, Extracellular matrix, Insulin, Insulin-secreting cells, Spleen

## Abstract

**Objective(s):**

Type 1 diabetes mellitus is a common autoimmune and multifactorial disorder. Researchers have been interested in making a favorable islet-like tissue model for the treatment of diabetes. The main objective of this study was to determine the effects of the spleen extracellular matrix (S-ECM) on the function of the MIN6 cell line (a β-cell model).

**Materials and Methods:**

In this experimental research, Wistar rat spleens were decellularized by sodium dodecyl sulfate (SDS) and Triton X-100. S-ECM was characterized by histological assessments, scanning electron microscopy, determination of residua DNA, and examination of the mechanical tensile property. Then, MIN6 cells were seeded on S-ECM scaffold. Glucose-stimulated insulin secretion and mRNA expression of insulin-related genes were examined to confirm the function of the cells.

**Results:**

The main components of S-ECM such as collagen and glycosaminoglycan remained after decellularization. Furthermore, very low residual DNA and appropriate mechanical behavior of S-ECM provided an ideal extracellular microenvironment for the MIN6 cells. GSIS results showed that the seeded cells in S-ECM secreted more insulin than the traditional two-dimensional (2D) culture. The expression of specific insulin-related genes such as PDX-1, insulin, Maf-A, and Glut-2 in the recellularized scaffold was more significant than in the 2D traditional cultured cells. Also, MTT assay results showed that S-ECM were no cytotoxic effects on the MIN6 cells.

**Conclusion:**

These results collectively have evidenced that S-ECM is a suitable scaffold for stabilizing artificial pancreatic islands.

## Introduction

Type 1 diabetes is a lifelong chronic autoimmune condition characterized by insulin deficiency due to the destruction of pancreatic β-cells by the immune system (1). Current treatments do not cure complications associated with the disease (2). Transplanting Langerhans islands or replacing β-cells are effective treatments for diabetes (3). However, the application of islet transplantation is limited by the following points: the need for long-time suppression of the immune system and the lack of donors (4).

The production of insulin secreted cells (ISCs) can solve the mentioned problems. However, a gradual decrease in insulin secretion has limited the transplanting of ISCs (5). Recent advances in tissue engineering have led to the development of scaffolds with a similar native extracellular matrix for seeding cells to tissue transplantation. Successful generation of extracellular matrix (ECM) scaffolds have been reported in several tissues such as the liver, nerve, respiratory tract, mammary gland, tendon, and bladder (6-9). ECM scaffolds are generated by various physical and enzymatic decellularization methods (10). Removing allogenic and xenogenic cells from tissue induces the most negligible immune response. Additionally, removing the cells provides an ideal three-dimensional (3D) structure for tissue regeneration (11).

The spleen tissue has glycoprotein**, **collagen**, **proteoglycan, ECM-affiliated protein, and blood vessel (12). The presence of multiple ECMs in the spleen provides a suitable model for tissue engineering (12). Several matrix molecules such as laminin, fibronectin, and collagen have contributed to forming interstitial matrices of the spleen tissue (13).

The mouse pancreatic β-cell line MIN6 is very similar to the β-cells, which is a suitable model for studying the mechanism of glucose-stimulated insulin secretion (14, 15). The MIN6 cells show increased insulin secretion and glucose sensitivity when placed in the soft scaffolds (16).

This study aimed to develop and characterize an islet-like tissue using S-ECM for preserving pancreatic β-cell function.

## Materials and Methods

***Decellularization of the spleen***

The Wistar rats were from the animal center of Ahvaz Jundishapur University of Medical Sciences. The experiment was done according to the Animal Welfare Act and was approved by the Animal Ethics Committee of the Ahvaz Jundishapur University (code: IR.AJUMS.ABHC.REC.1400.012). The spleen of the healthy Wistar rats under deep anesthesia was removed and stored at -70 °C for 24 hr. 

The excessive tissues around the spleens were removed, and the samples were cut into small pieces (0.5 cm^3^). The tissue fragments were subjected to 1% sodium dodecyl sulfate (SDS) in phosphate-buffered saline (PBS) and vibrated for 72 hr, followed by treatment with 1% Triton X-100 solution for 30 min to remove the SDS detergent thoroughly. The SDS solution was replaced three times every 24 hr. Finally, the spleens were washed with PBS for 60 min and dried at room temperature. 

***Histological evaluation of S-ECM***

For the cross-sectional observations, the native and decellularized spleens were fixed in 10% formalin, paraffin-embedded, and cut into sections (5 μm).

The sections were stained with hematoxylin and eosin (H & E), Masson’s trichrome, alcian blue, and DAPI (4′,6-diamidino-2-phenylindole) [Sigma-Aldrich, USA]. Quantitation of the staining intensity was measured as a percentage of the total area using Image J software (National Institutes of Health, Bethesda, USA).

***Determination of residual DNA***

The DNA content of the decellularized and native tissues was extracted by the saline extraction method (17). The tissues were lyophilized, cut into small pieces, and incubated in extraction buffer (1 M Tris, 5 M NaCl; 0.5 M EDTA, pH = 8) and lysis buffer (extraction buffer containing proteinase K and 10% SDS) overnight. The DNA content of the digested tissues was precipitated by 5 M NaCl. The precipitated proteins were removed, and the remaining DNA was diluted in 100 μl distilled water. The total amount of DNA was determined by a NanoDrop spectrophotometer.

*Mechanical tensile test*

The mechanical behavior of S-ECM was evaluated in wet (cultivated in culture media for ten days) and dry conditions by the material device (Wance, China) equipped with a 5 kilonewtons (kN) load cell. At first, strip-shaped pieces were prepared at a five mm width˟10 mm length from the samples. The samples were then clamped in device grips. Tensile test and their analysis were performed after setting the crosshead speed at 5 mm/min.

***Scanning electron microscopy (SEM) analysis***

The seeded and unseeded scaffolds were fixed with 2.5% Glutaraldehyde in PBS (pH 7). After two washes with PBS (pH 7), the samples were fixed in 1% osmium tetroxide. Dehydration was done with successive ethanol treatments before critical‐point drying with liquid carbon dioxide. 

The surface of the samples was sprayed with gold and loaded into a scanning electron microscope platform (Hitachi, Japan) to observe the surface morphology of the scaffolds.

***Recellularization of S-ECM***

MIN6 cell line were bought from the Type Culture Collection of the Iranian Biological Research Center (Tehran, Iran). The MIN6 cells were cultured in Dulbecco’s Modified Eagle’s Medium (DMEM) containing 10% fetal bovine serum (FBS), 50 μM β-mercaptoethanol, and 100 U/ml penicillin/streptomycin at 37 °C and 5% CO_2_ atmosphere. S-ECM scaffolds were sterilized with 70% ethanol and kept on a 24-well culture plate. After soaking the scaffolds in the complete medium for 24 hr, the MIN6 cells (1×10^4^) were suspended in 100 μl culture medium and seeded on the scaffolds, followed by adding one milliliter of culture medium per well. 

The recellularized scaffolds were placed in a standard cell culture incubator at 37 °C, and the media were changed every two days. At 70–80% confluency, EDTA (100 mM) was added, and the cells were collected.

***Cell cytotoxicity using the MTT assay***

S-ECM scaffold’s biocompatibility was investigated by viability measurement of the MIN6 cells using an MTT assay. The cultured cells on the wells without scaffolds were used as the control. In brief, 1×10^4^ cells/well were seeded on S-ECM scaffolds in the 24-well plates. DMEM were removed, and MTT solution (5 g/l) was added to each well and incubated in the dark for 4 hr. 

After 4 hr, the medium was removed and replaced with one ml DMSO to dissolve formazan and incubated in the dark for 10 min. Finally, the absorbance was read at 570 nm using a microplate spectrophotometer (Epoch, BioTek). 

***Glucose-stimulated insulin secretion***

Glucose-stimulated insulin secretion (GSIS) assay was performed on S-ECM containing MIN6 cells and two-dimensional (2D) cell culture. MIN6 cells were treated with Krebs Ringer Bicarbonate buffer (KRBH; 4.7 mmol/l KCl + 115 mmol/l NaCl + 1.2 mmol/L KH_2_PO_4_ + 20 mmol/l NaHCO_3_ + 1.2 mmol/l MgSO_4_. 7 H_2_O + + 2.56 mmol/l CaCl_2_ + 0.2% bovine serum albumin) for 2 hr without glucose. The cells were then exposed to the KRBH with low glucose (2 mmol/l) or high glucose (20 mmol/l) for 1 hr, and the supernatant was collected. The supernatants were stored at – 80 °C before insulin analysis by a mouse ELISA kit (Mercodia, Sweden).

***Quantitative real-time PCR analysis***

The total cell RNA were isolated with the RNeasy Mini Kit (Qiagen, Valencia, CA) based on the manufacturer’s protocol. Then, reverse transcription reactions were done with one microgram of total RNA and PrimeScript™ RT reagent kit (Takara). SYBR premix ExTaq (Takara Bio, Shiga, Japan) was applied to real-time RT-PCR using an ABI one-step system with specific primers. The following primers were used in this study: CAGCTGTCTTGTGCTCTGCTTGT (Forward Glut-2), GCCGTCATGCTCACATAACTCA (Reverse Glut-2); AGGCCTTCCGGGGTCAGAG (Forward Maf-A), TGGAGCTGGCACTTCTCGCT (Reverse Maf-A); GGACGACCCGATGAGTCT (Forward PDX-1), TGCTGGGCACCAGTTCCTT (Reverse PDX-1); AGCGTGGCTTCTTCTACACAC (Forward insulin), CTGGTGCAGCACTGATCTACA (Reverse insulin); GTCTCCTCTGACTTCAACAGCG (Forward GAPDH), ACCACCCTGTTGCTGTAGCCAA (Reverse GAPDH). The following 45-cycle program were used to amplify the PCR: 95 °C for 10 sec, 95ºC for 15 sec, 60 °C for 20 sec, and 60 °C for 20 sec. The relative expression were calculated by the 2^-ΔΔCt^ method and normalized by GAPDH.

***Statistical analysis***

Data were analyzed in *SPSS* (version 22.0, *USA*) using one-way analysis of variance, followed by a *pos thoc* pairwise comparison applying Tukey or LSD tests or Kruskal-Wallis for non-parametric data. The *P*-values<0.05 were statistically significant. Each assay, at least, was repeated three times.

**Figure 1:**
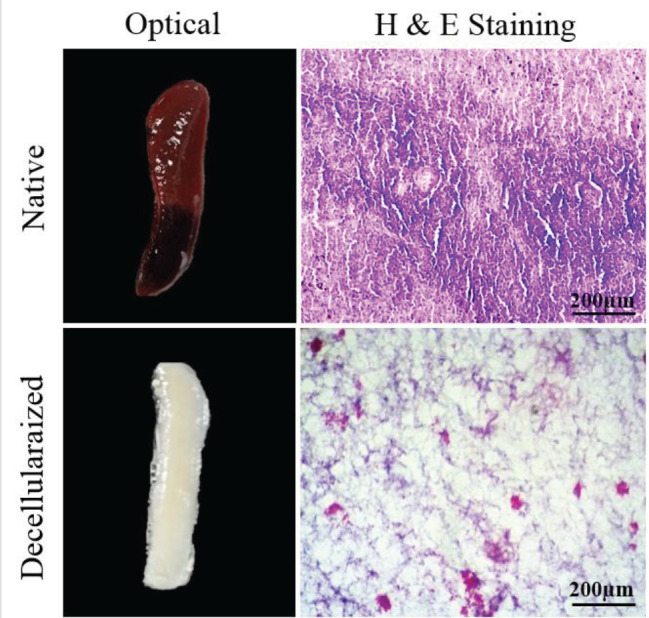
Optical and microscopic images (H & E staining) of the native and decellularized spleen. Decellularized tissue has a transplant appearance and a few nuclei

**Figure 2:**
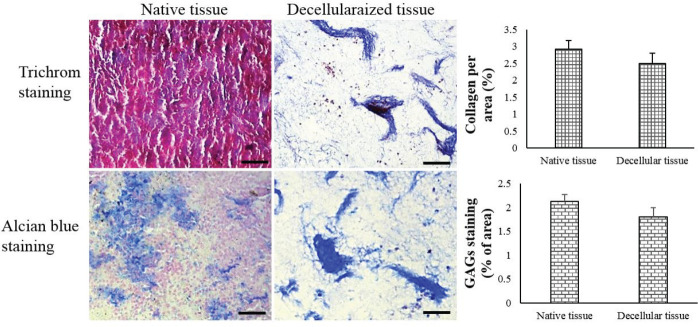
Microscopic images of the native and decellularized spleens stained by Trichrome Masson and Alcian Blue. The percentage of collagen and GAGs per area obtained by ImageJ software were also reported (mean ± SD)

**Figure 3:**
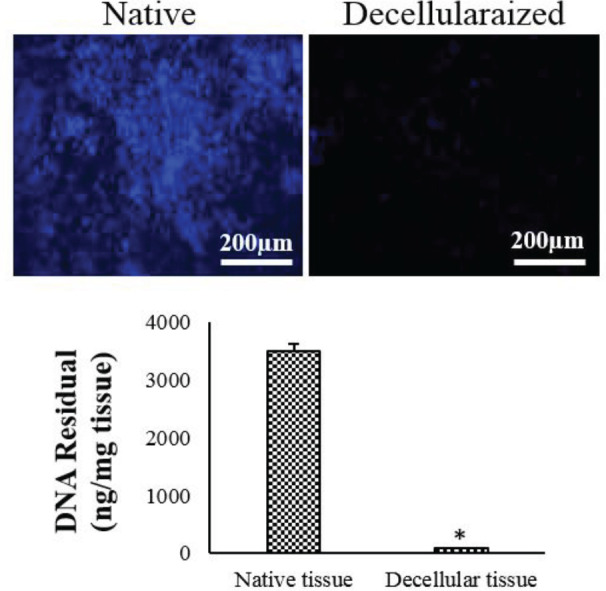
Fluorescence images of DAPI staining show a few nuclei in the decellularized spleen tissue. The amount of DNA in the native and decellularized spleen have also been illustrated (mean ± SD), * *P*<0.001

DAPI: 4′,6-Diamidino-2-phenylindole

**Figure 4:**
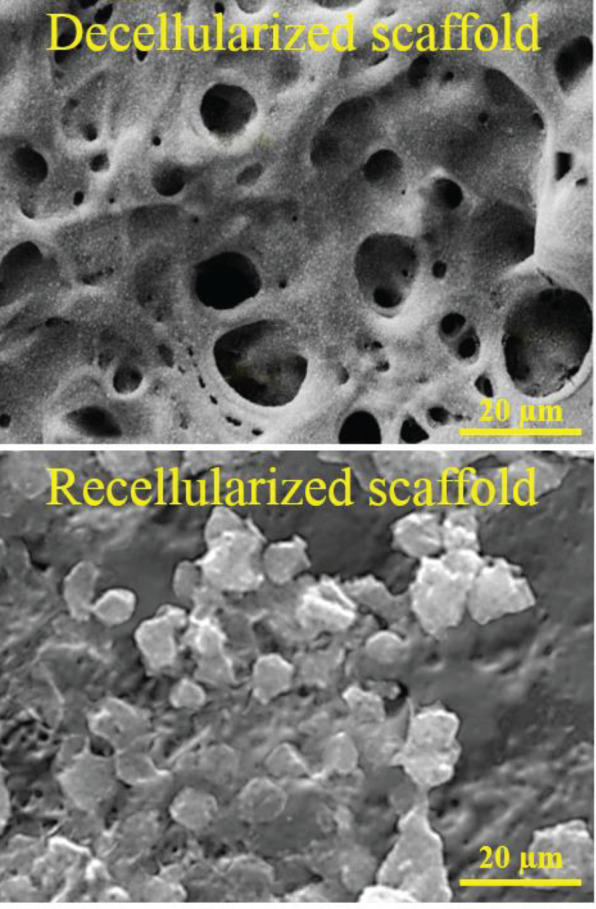
SEM micrographs of seeded and unseeded S-ECM show the cells removed from the splenic tissue after decellularization. The MIN6 cells aggregated and formed cell clusters in S-ECM

SEM: Scanning electron microscopy; S-ECM: Spleen extracellular matrix

**Figure 5:**
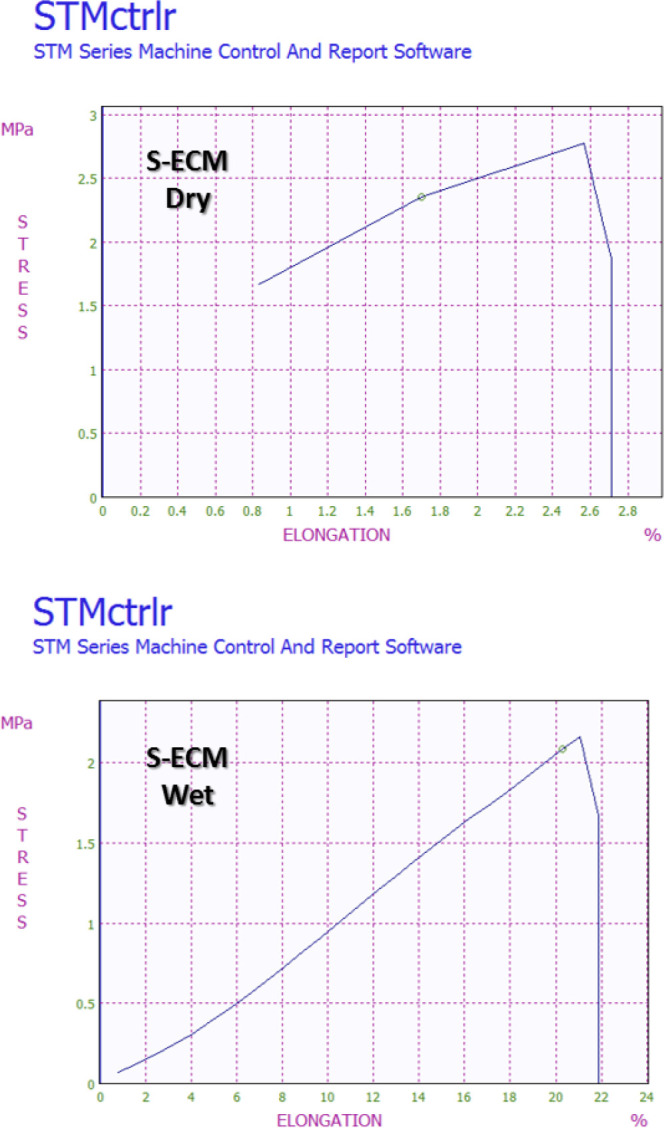
The results of the mechanical tensile test (Stress-strain data) of the S-ECM at wet and dry conditions

S-ECM: Spleen extracellular matrix

**Figure 6:**
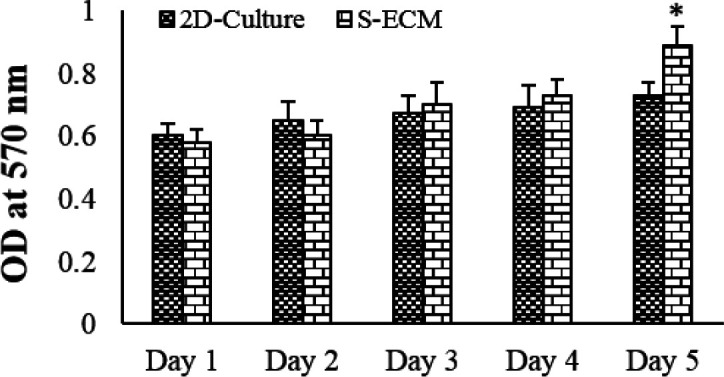
Cytotoxicity assessment of the scaffolds against MIN6 cell line after different durations of cell culture (mean ± SD), * *P*<0.001

**Figure 7:**
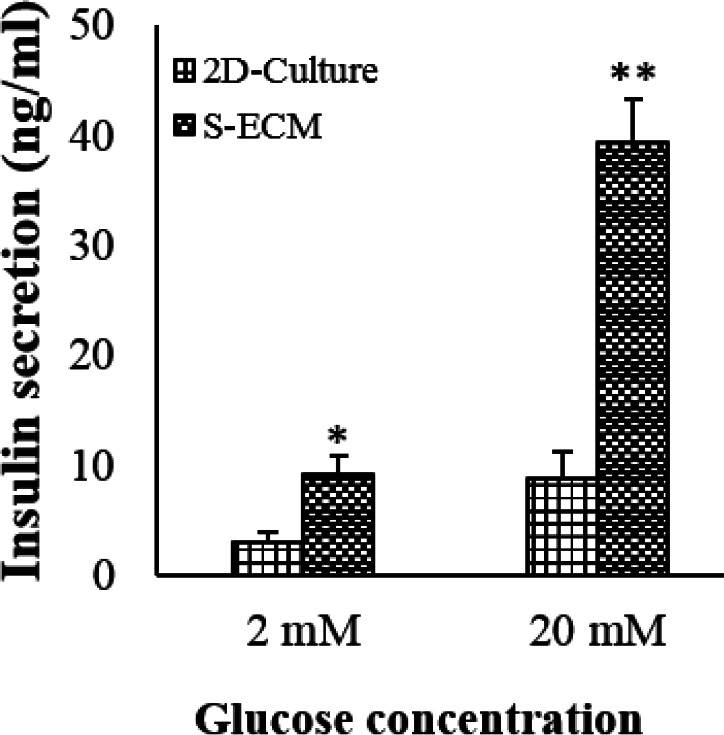
Insulin secretion of the MIN6 cells in response to low and high glucose stimulations (mean ± SD). *P<0.01, *P*<0.001. * indicates comparison with the 2D-culture cells

**Figure 8:**
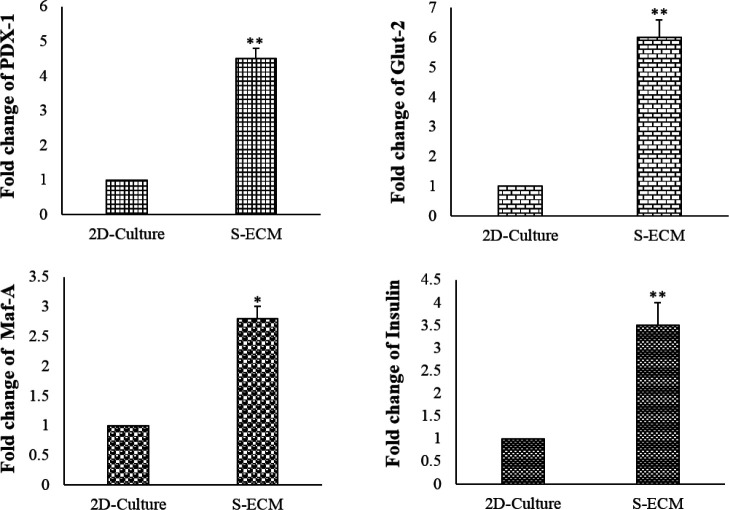
Expression of PDX-1, Maf-A, Insulin, and Glut-2 genes in S-ECM and 2D-cultured cells. Bar graph showing the significant difference between the two system cultures (mean ± SD), * *P<*0.05, **P<0.01

S-ECM: Spleen extracellular matrix

## Results

***Histological analysis***

Approximately 72 hr after the beginning of the decellularization process, the spleens were completely translucent (Figure 1). H & E staining revealed that the nuclear and cytoplasmic materials had been deleted from S-ECM after decellularization (Figure 1). The tricolor Masson staining confirmed that there was no considerable loss of collagen in S-ECM scaffold (Figure 2). The maintenance of glycosaminoglycans (GAGs) in S-ECM scaffold were demonstrated by Alcian Blue staining (Figure 2). DAPI staining showed no nuclear residues in the decellularized splenic tissue, and its DNA content was noticeably lesser than the intact tissue (Figure 3). 

SEM analysis of the scaffold revealed that the decellularized spleens were free of cells leaving only spaces left empty by the solubilized cells (Figure 4).

***Residual DNA assessment***

As illustrated in Figure 3, the residual DNA content of the decellularized scaffolds was significantly lower compared with the native splenic tissue (*P*<0.001). 

***Mechanical characterization***

Dry and wet samples were subjected to a uniaxial tensile test. The stress-strain results displayed a typical hyper-elastic response of biological soft tissue. The dry scaffold exhibited a higher maximum load compared with the wet scaffold.  Maximum elongation in strain was significantly higher in the wet scaffold compared with the dry scaffold. Statistically significant differences in maximum force, modulus and elongation values at maximum force were observed. These results were illustrated in Figure 5.

***Cell viability***

The viability of S-ECM-seeded cells was not significantly changed compared with the 2D-traditional culture at different times. However, the viability of S-ECM-seeded cells on the fifth day was significantly more than on the first day (*P*<0.05; Figure 6). 

***Insulin release***

The GSIS results evidenced that under high or low glucose, the MIN6 cells seeded in S-ECM could secret more insulin in response to glucose stimulation than the 2D-cultured cells. These results were depicted in Figure 7.

***Gene expression***

The impacts of S-ECM on the expression of PDX-1, insulin, Maf-A, and Glut-2 genes were analyzed. S-ECM could significantly increase the mRNA expression of PDX-1 (2.5-fold), Maf-A (1.8-fold), insulin (2.2-fold), and Glut-2 (1.4-fold) in the MIN6 cells in comparison with the 2D-cultured cells (Figure 8).

## Discussion

In the current study, decellularized spleen as a scaffold was used for seeding MIN6 cells to establish an artificial pancreatic islet. The splenic tissues were decellularized using the detergent ionic SDS and Triton X-100. SDS is an effective detergent for decellularization that retains the 3D structure of ECM (18). However, at high concentrations, it harms recellularization. Hence it is essential to remove the SDS residue from the scaffold. In this study, Triton X-100 was used to remove unbounded SDS from proteins. Maintaining the structure and composition of ECM is one of the essential requirements for the normal cellular behavior of scaffold recellularization (19, 20). Our decellularization protocol removed cellular elements and DNA residue and kept the structure of the splenic tissue. Removing residual DNA and cell debris leads to better-placed cells in the scaffold, decreases the immune response, and increases the survival of the seeded cells (21). The main components of ECM containing collagen and GAGs remained in S-ECM.  The selected scaffold must have mechanical properties close to the desired tissue. Therefore, the mechanical properties of S-ECM were evaluated with the tensile test. As revealed in the results, S-ECM scaffold had appropriate tensile strength and elongation. 

The seeded MIN6 cells in S-ECM scaffold could aggregate and forms cell clusters similar to other studies (22-23). The clustered-β-cells can release more insulin than the single cells (22, 24). The seeded MIN6 cells in S-ECM not only generated insulin but also could significantly secrete insulin in response to glucose stimulation. This finding evidenced that S-ECM promotes MIN6 function. In a previous study, a hydrogel scaffold with tunable microenvironmental properties could enhance insulin secretion from the MIN6 cells (25). Furthermore, Chaimov *et al*. (26) found that hepatocytes trans-differentiated to β-cells on pancreas-ECM induced an over 4-fold increase in insulin secretion. In this study, the seeded scaffold showed a 4.4-fold increase in insulin secretion. The reasons for higher insulin secretion from S-ECM-cultured cells might be due to the following points: nutrients and cellular metabolism can considerably affect glucose-stimulated insulin secretion. S-ECM culture systems were completely immersed in culture media for hr before use so the cells could get more nutrients from S-ECM compared with the plastic surface of the 2D culture. 

To confirm the improvement of MIN6 function by S-ECM, the mRNA expression of conventional insulin-trans-activating genes was analyzed. The expression of Glut-2, insulin, PDX-1, and Maf-A in S-ECM-seeded cells was higher than in the 2D-cultured cells.  In the β-cells, glucose uptake is regulated by Glut-2, which is essential for insulin secretion in response to glucose (27). MIN6 cells, in response to glucose stimulation, increase the expression of Glut-2 (14, 15). The interaction of PDX-1 with Glut-2 and Maf-A is crucial for activating insulin gene transcription (28). The PDX-1 inactivation of the β-cells leads to diabetes in mice (29). Maf-A binds to a conserved insulin enhancer element RIPE3b/C1-A2 and activates insulin gene expression (30-31). The enhancing expression of the specific insulin-related genes confirms the improvement of MIN6 cell function by S-ECM. In line with our results, previous *in vitro* and *in vivo* studies of MIN6 cells in decellularized pancreas showed up-regulating pancreatic insulin expression (23). Park *et al*. investigated the effect of splenocytes on β-cell function and mass in type 2 diabetic rats with and without spleen.  They reported that splenectomy increased and sustained serum glucose levels during the oral glucose tolerance tests. At the same time, splenocyte injection increased β-cell neogenesis and function (32).

As revealed in the results, S-ECM scaffold was nontoxic to MIN6 cells, and the cells could grow and proliferate on S-ECM scaffold. Cytocompatibility studies have indicated that the decellularized scaffolds obtained from the pancreas, liver, and spleen have no toxic impacts on the seeded cells (12, 33-35). When the MIN6 β-cells were perfused into the mouse pancreas-ECM, Goh *et al*. (36) found cell survival and maintenance of insulin gene expression after 5 days. Among ECM-macromolecules, laminins and collagens can enhance the survival of the islets (37). Laminin may increase islet survival by enhancing the expression of PDX-1, insulin, glucagon, and Glut-2 (38). The spleen tissue has various ECM-affiliated proteins, glycoproteins, collagens, and proteoglycans (12). Interestingly, islet-ECM contains collagen (types I, III, IV, V, and VI), laminins, and fibronectin (39, 40).  Laminin and fibronectin are associated with β-cell proliferation, differentiation, and insulin secretion (41, 42). ECM-proteins such as fibronectin and laminin enhance pancreatic differentiation with increasing expressions of the Glut-2 and insulin genes, insulin protein levels, and insulin release in response to glucose stimulation (42). An artificial hydrogel scaffold containing collagen IV and laminin provided an ideal microenvironment for insulin secretion of the MIN6 cells (43, 44). Collagen VI supports islet-cell viability and improves insulin secretion of human pancreatic islets (45). 

Although in the present study potential impact of S-ECM on β-cell regeneration or neogenesis of β-cells was not studied, high expression of the β-cell-specific genes such as PDX-1 and Maf-A indicate that S-ECM promotes β-cell regeneration. In addition to the natural roles of these genes in β-cell maturation, ectopic expression of Maf-A and PDX-1 has been successfully used to reprogram various cell types into insulin-secreting cells *in vivo* and *in vitro* (46). In addition, PDX-1 is involved in β-cell neogenesis in various models of pancreas regeneration (47, 48). There are reports in the literature that indicate S-ECM is suitable for tissue regeneration (35, 49). Xiang *et al*. have reported that S-ECM improves the differentiation of bone marrow mesenchymal stem cells into functional hepatocyte-like cells (35).

## Conclusion

The present study demonstrated a simple protocol for preparing a spleen scaffold with minimal damage toECM and its components as a novel 3D cell culture platform. S-ECM scaffold could enhance β-cell function by increasing the expression of insulin-specific genes and elevation of insulin release in response to glucose stimulation. The decellularized spleen can be considered an ideal substrate for β-cell transplantation. 

## Authors’ Contributions

YAF and LK Participated in study design, data collection and evaluation, drafting, and statistical analysis. YAF, LK, FN, and AHM Contributed to all experimental work such as spleen decellularization, histological assessment, DAPI staining, cell culture, data and statistical analysis, and interpretation of data. YAF, MO, and DBN Conducted molecular experiments and RT-qPCR analysis. YAF Drafted the manuscript, which was revised by LK and MO. All authors performed editing and approved the final version of this paper for submission, participated in the finalization of the manuscript, and approved the final draft.

## Conflicts of Interest

The authors declare no conflicts of interest. 
